# Feminizing the Face: Combination of Frontal Bone Reduction and Reduction Rhinoplasty

**DOI:** 10.1155/2018/1947807

**Published:** 2018-07-02

**Authors:** C. J. Salgado, H. AlQattan, A. Nugent, D. Gerth, W. Kassira, C. S. McGee, L. Wo

**Affiliations:** Division of Plastic, Reconstructive, Transgender, and Aesthetic Surgery, University of Miami Leonard M. Miller School of Medicine, Miami, FL, USA

## Abstract

Gender affirmation surgeries in male-to-female patient transitioning include breast augmentation, genital construction, and facial feminization surgery (FFS). FFS improves mental health and quality of life in transgender patients. The nose and forehead are critical in facial attractiveness and gender identity; thus, frontal brow reduction and rhinoplasty are a mainstay of FFS. The open approach to reduction of the frontal brow is very successful in the feminization of the face; however, risks include alopecia and scarring. Endoscopic brow reduction, in properly selected patients, is minimally invasive with excellent outcomes avoiding these risks. Since both reduction rhinoplasty and frontal brow reduction are routinely performed in FFS, a combined approach provides superior control over the nasal radix and profile when performing surgery on the frontal bone region first followed by nose reduction. We present a case series of four transwomen undergoing frontal bone reduction in combination with a reduction rhinoplasty. All had excellent results with one DVT that resolved with treatment. Transgender patients frequently require multiple operations during their transition increasing their hospital stay and costs. This combined approach offers superior control over the nasofrontal angle and is not only safe but reduces hospitalizations and costs and is a novel indication to reduce gender dysphoria.

## 1. Introduction

With increasing coverage of medically necessary procedures for transgender patients by health insurances and major industries, more patients have benefited from a decrease in their dysphoria and ultimately their quality of life. Gender affirmation surgeries in male-to-female transition patients include breast augmentation, genital construction, and facial feminization [1]. Facial features serve an integral role in identifying an individual's gender, with certain characteristics perceived as feminine or masculine [2]. The approach to analysis and planning of facial feminization surgery involves division of the face into the upper, middle, and bottom thirds. Feminization of the bottom third of the face can be addressed by procedures to decrease the angle of the mandible, lip augmentation through the use of fillers, and narrowing of the chin via genioplasty. The nose, as the centerpiece of the middle third of the face, is an important component of facial attractiveness and gender identity. Thus, reduction rhinoplasty is a mainstay of facial feminization surgery, while cheek augmentation and widening of the zygomatic complex can be employed to achieve a more feminine heart-shaped contour of the face [3]. The upper third of the face is considered to be the most significant portion in recognizing female gender. The male brow and forehead are typically more pronounced, especially in the glabella and superior orbital rim regions [2–4]. The open approach via a bicoronal incision or a pretrichial incision for exposure of the frontonasal-orbital complex has been the most common approach for modification of this bone. Patient satisfaction rates are high with open forehead contouring procedures, but open approaches impart risks such as alopecia, scarring, paresthesias, and, rarely, damage to the frontal branches of the facial nerve [5–7].

Endoscopy has been used in craniofacial surgery for the last 20 years primarily in the trauma setting for fracture reduction [8]. Endoscopic-assisted facelifts and forehead lifts have also recently been described as well [9]. Guyuron et al. published a series on endoscopic reduction of frontal bossing in 10 patients, with excellent aesthetic outcomes and no postoperative complications at follow-up [10].

Having performed both modification of the frontal bone and reduction rhinoplasty individually in this patient population, we noticed that when using a combined approach, performing the brow reduction prior to the reduction rhinoplasty, one is allowed superior control over the nasal radix and profile of the nose in one stage. We present a case series and our technique in transgender patients undergoing either open or endoscopic frontal bone reduction in combination with a reduction rhinoplasty for facial feminization.

## 2. Materials and Methods

### 2.1. Surgical Technique: Endoscopic Brow Reduction

**Figure 1:**
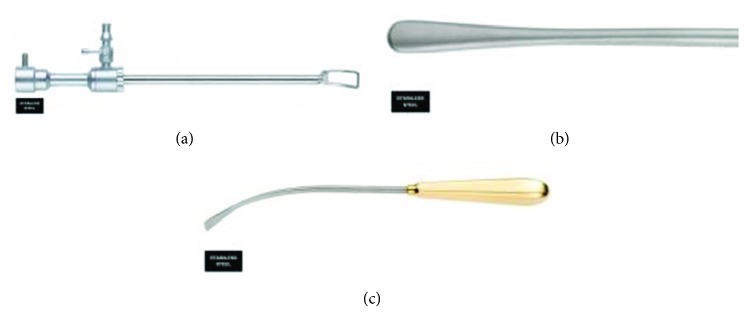
(a) Endoforehead fenestrated cannula. (b) Endoscopic straight periosteal elevator. (c) Endoscopic curved periosteal elevator.

**Figure 2:**
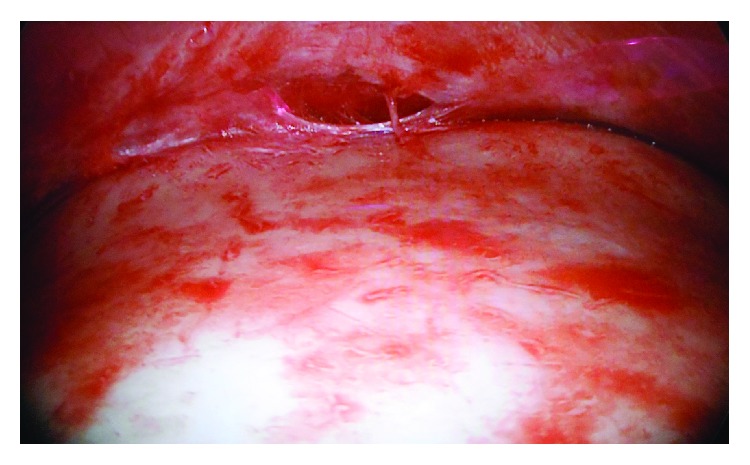
Intraoperative endoscopic view.

Following a preoperative lateral cephalogram, an assessment is made of the anterior table of the frontal sinus. Preoperative facial bone CT provides significantly more detail on the anatomy of the glabellar region and the possible varying thickness of the anterior table in this region. Patients with an anterior table thickness of greater than 5 mm are amenable to burring with a notable difference in their facial aesthetics. These are candidates for an endoscopic brow procedure, particularly, if a hairline lowering procedure is not needed. A 30-degree 4 mm endoscope attached to an endoforehead fenestrated cannula (Figure 1(a)) and straight and curved periosteal elevators (Figures 1(b) and 1(c)), as well as an angled Midas Rex high-speed surgical drill (Medtronic), attached to a round cutting burr are the typical instruments used in the endoscopic brow reduction technique. We perform these cases under general anesthesia using an oral RAE tube with the patient's occiput positioned in a Mayfield horseshoe headrest. This allows for optimal exposure and access to the surgical site. For the endoscopic access ports, we place two incisions approximately 1-2 cm posterior to the frontal hairline in an anteroposterior direction, each in the paramedian position equidistant from each other. Once the endoscope and fenestrated cannula is introduced under the flap, we dissect anteriorly in a subgaleal and then subperiosteal plane to approach the supraorbital rim. Care must be taken not to violate the supraorbital nor supratrochlear pedicles during this dissection. The most prominent area of protuberance is typically just superior to the glabellar region in the paramedian plane. We then proceed to burr the anterior table of the frontal sinus with numbers 4 and 3 cutting burrs, followed by a number 3 diamond burr for more refined contouring at 20,000 rpm. An irrigating tube is used to prevent thermal injury to the bone. Once the underlying bone becomes translucent, the endpoint is reached and no further reduction can be performed without entering the frontal sinus cavity. Constant motion of the drill and avoidance of aggressive drilling 10–20 mm lateral to the midline help to minimize the risk of exposure of frontal sinus mucosa as these areas of bone tend to be thinner (Figure 2) [11].

Once sufficient contouring has been achieved, assessment of the eyebrow position is performed, and if a brow lift is indicated, forehead skin can be removed accordingly. We perform a concurrent septorhinoplasty, usually with a second surgical team. Dorsal nasal hump reduction, nasal bone osteotomies, submucosal septal cartilage resection, nasal tip refinement via cephalic trimming, and columellar strut grafts are standard in our technique. Turbinate outfracture, spreader grafts, and lateral crural strut grafts are performed as needed, depending on the patient's nasal airway. A soft-pressure dressing is applied and maintained in position for 24 hours, along with head elevation while at rest for 3 days to minimize associated edema.

### 2.2. Surgical Technique: Open Brow Reduction

Following a preoperative lateral cephalogram, an assessment is made of the anterior table of the frontal sinus. Patients with an anterior table thickness of greater than 5 mm are amenable to burring with a notable difference in their facial aesthetics. If a hairline lowering procedure is also needed, then these are candidates for an open approach. A pretrichial incision extending across both temporal areas and the midline is marked and infiltrated with a local anesthetic and epinephrine. Our incision is slightly beveled anteriorly, and subgaleal dissection is carried out to the midportion of the forehead where we then transition to a subperiosteal dissection. Periosteal elevators are used to elevate the periosteum and create a pericranial flap that extends to the supraorbital rim to be used if needed. We extend the dissection to include the lateral orbital wall to obtain a clear view of the frontal bone with unhindered access. The supraorbital and supratrochlear neurovascular bundle must be identified and preserved during this dissection. The areas of most protuberance are identified and burred down as previously described in the endoscopic brow reduction technique.

If the patient's anterior table is too thin for burring (less than 5 mm), then a frontal bone setback is performed. We drill circumferentially around the frontal sinus, and the anterior table of the frontal bone is removed as two main segments due to their fusion in the midline. The intersinus septum and midline air cells are divided. The anterior table segments are transferred to a back table where they are further contoured using a burr and then plated to a titanium mesh. Simultaneously, the remaining supraorbital rim and periorbital contour is further refined using burring techniques. Finally, the anterior plate segments are secured to the margin of the frontal sinus using 4-5 mm screws. A scalp reduction is then performed by undermining the posterior incision in the subgaleal plane and advancing the scalp flap anteriorly to lower the hairline. The excess skin is excised off the forehead flap. The soft tissue of the brow and galea is secured with suture to the titanium mesh to create a temporal brow lift bilaterally. The scalp incision is closed in layers with absorbable sutures.

To evaluate postoperative success, patients were asked to qualitatively rate their satisfaction as ranging from “not satisfied,” “satisfied,” and “very satisfied” at follow-up. We saw every patient in the follow-up clinic, with a mean follow-up of 21 months.

## 3. Results

### 3.1. Case 1

**Figure 3:**
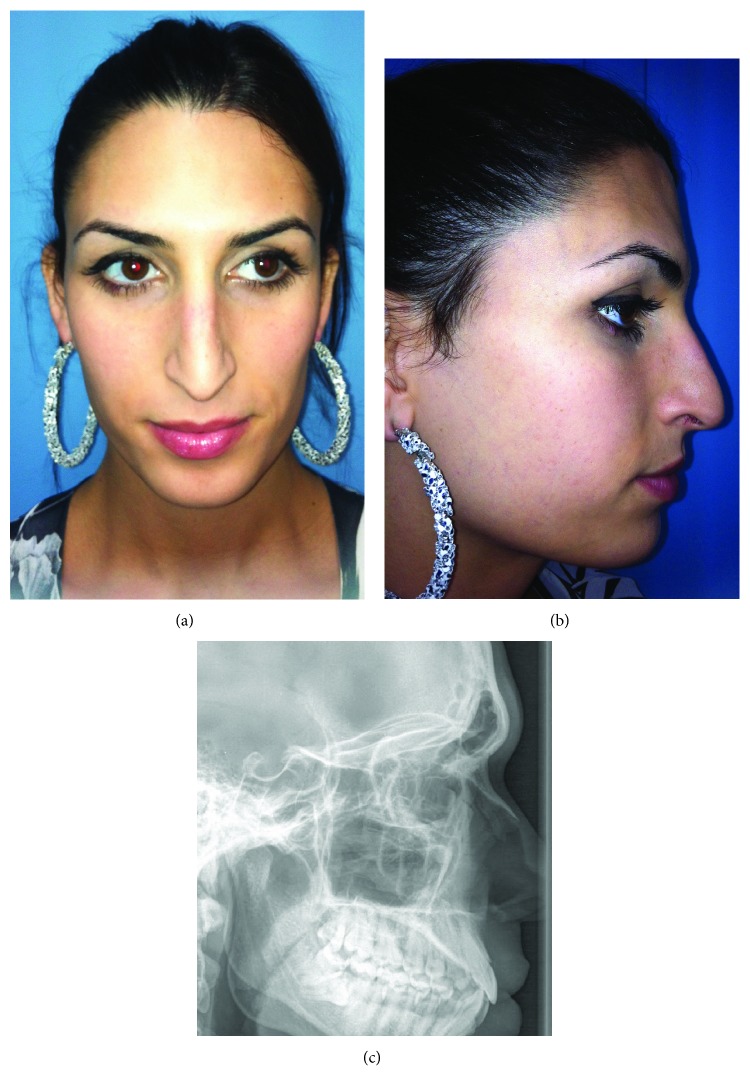
(a) Preoperative frontal view. (b) Preoperative right lateral view. (c) Preoperative right lateral cephalogram demonstrating a thick anterior table.

**Figure 4:**
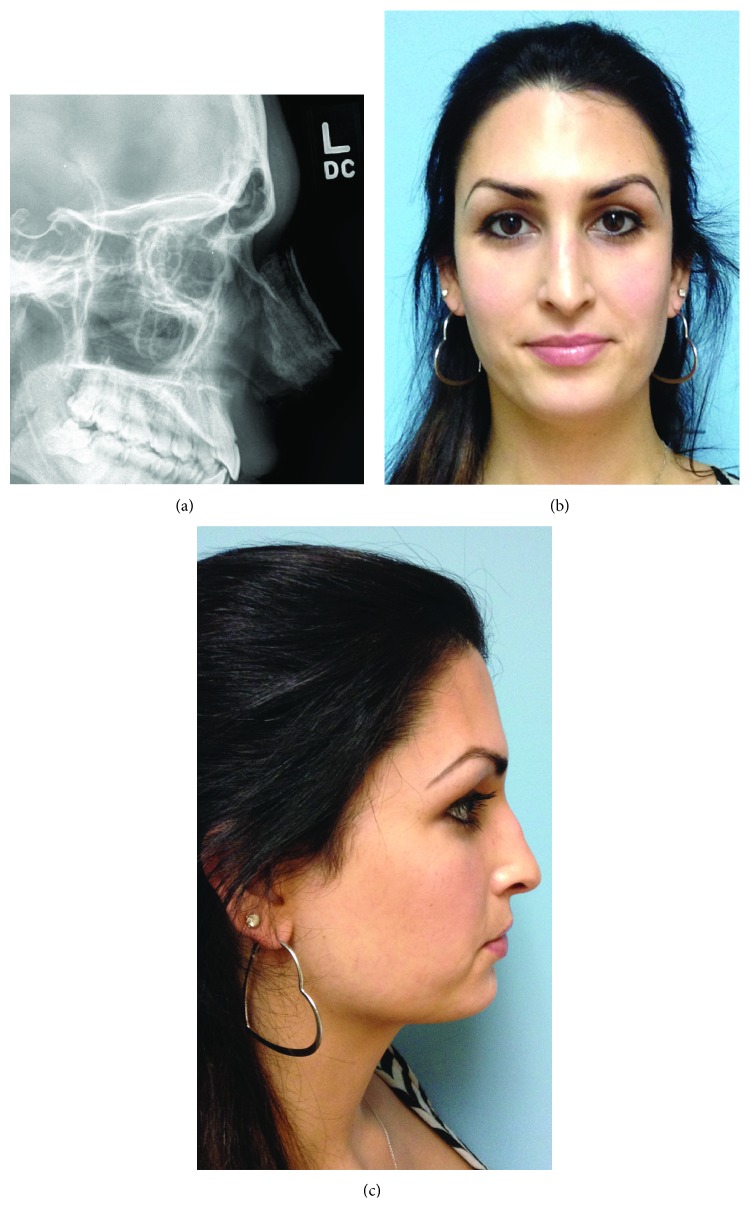
(a) Postoperative right lateral cephalogram. (b) Postoperative frontal view at 2 years. (c) Postoperative right lateral view at 2 years.

A 27-year-old male-to-female transgender patient presented to our clinic desiring a more feminine face. She had previously undergone a rhinoplasty and was specifically concerned with her brow and residual nasal asymmetry. On physical exam, she had a prominent superior orbital rim, right nasal septum deviation, a dorsal hump, and airway obstruction on the right side with a droopy tip (Figures 3(a) and 3(b)). Preoperative lateral cephalograms confirmed a thick anterior table of the frontal sinus making her an ideal candidate for endoscopic reduction (Figure 3(c)). Three weeks postoperatively, she was seen in the clinic with a significant improvement in the supraorbital bossing and a softer more feminine nose. Postoperative lateral cephalograms demonstrated thinning of the supraorbital prominence with a less acute angle at the superciliary arches (Figure 4(a)). At 3-month, 2-year, and 4-year follow-ups, she developed no complications, had an excellent aesthetic result, and was satisfied with the procedure (Figures 4(b) and 4(c)).

### 3.2. Case 2

**Figure 5:**
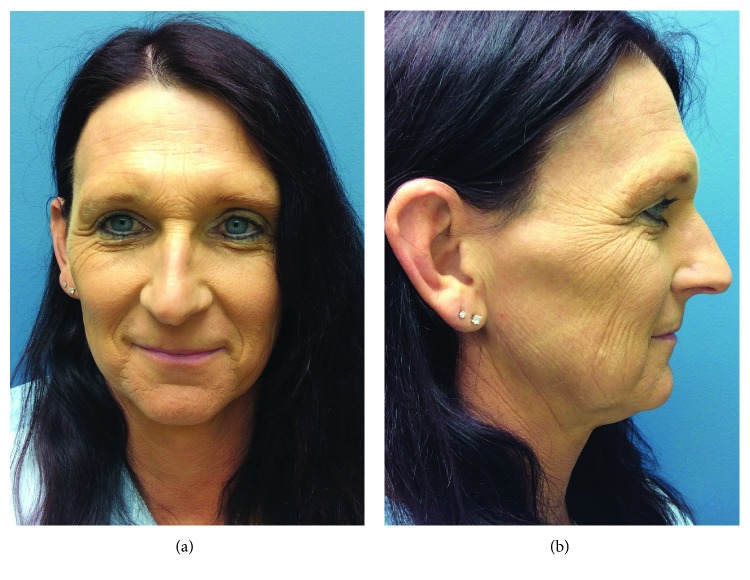
(a) Preoperative frontal view. (b) Preoperative right lateral view.

**Figure 6:**
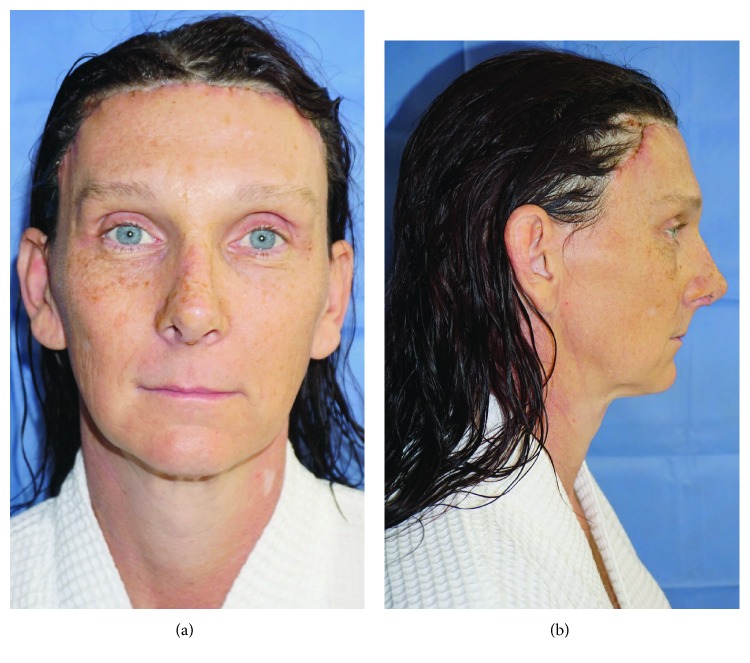
(a) Postoperative frontal view at 2 weeks. (b) Postoperative right lateral view at 2 weeks.

A 41-year-old male-to-female transgender patient presented to our clinic inquiring about facial feminization and gender confirmation bottom surgery. On examination of her face, she had a prominent supraorbital ridge, brow descent with wrinkling of the forehead, and a large masculine nose (Figures 5(a) and 5(b)). Preoperative lateral cephalograms confirmed a thick anterior table amenable to burring. Open frontal bone burring, frontal brow lift, and rhinoplasty were performed. At 2-week follow-up, she had excellent healing with pleasing results (Figures 6(a) and 6(b)) and postoperative lateral cephalograms demonstrated adequate thinning of the supraorbital prominence. She also underwent gender confirmation bottom surgery during the same hospitalization, 9 days prior to her facial feminization surgery. Her hospitalization was complicated by bilateral upper extremity DVTs which further work-up revealed Factor V Leiden. She was treated with a three-month course of anticoagulation with no long-term complications. At 8- and 16-month follow-ups, she continues to have no complications and is very satisfied with her results.

### 3.3. Case 3

**Figure 7:**
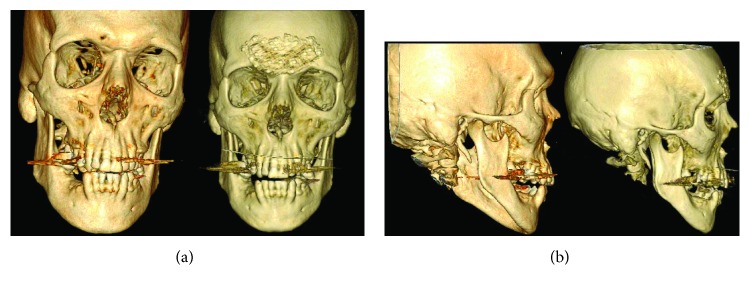
(a) Preoperative and postoperative anterior view of facial bones using CT with 3D reconstruction. (b) Preoperative and postoperative right lateral view of facial bones using CT with 3D reconstruction.

A 62-year-old male-to-female transgender patient presented to our clinic desiring a more feminine face. She had previously undergone gender confirmation bottom surgery with penile skin inversion in addition to hormonal therapy. Her areas of main concern were frontal bossing and the asymmetry of her nose. On physical exam, she had right nasal septum deviation with left-sided internal nasal valve collapse and a bulbous tip. Additionally, she reported a history of left nasal obstruction and recurrent sinus infections. CT of the preoperative facial bones confirmed a thin anterior table of the frontal sinus making her not suitable for an endoscopic approach. Open frontal bone set back with septorhinoplasty was planned. One week postoperatively, she was seen in the clinic for the removal of her intranasal and extranasal splints. CT of the postoperative facial bones with 3D reconstruction demonstrated a less acute angle at the superciliary arches with an anterior table set back (Figures 7(a) and 7(b)). Follow-ups at 3 and 10 months demonstrated excellent results with no complications and patient satisfaction.

### 3.4. Case 4

A 28-year-old male-to-female transgender patient presented to our clinic desiring a more feminine face. She had previously undergone gender confirmation bottom surgery with penile skin inversion in addition to breast augmentation and hormonal therapy. Her greatest areas of concern were the brow, nose, and chin. On examination of her brow, she had eyebrows at the level of the superior orbital rim with supraorbital bossing. Her nasal tip was slightly bulbous with a wide dorsum in addition to a bony prominence in the symphyseal and parasymphyseal regions. Preoperative lateral cephalogram confirmed a thick anterior table of the frontal sinus making her a suitable candidate for endoscopic reduction. Rhinoplasty in combination with endoscopic frontal bone reduction and transoral mandibular osteoplasty was performed. Follow-up at two weeks confirmed excellent wound healing, and her intranasal and extranasal splints were removed. She was very satisfied with her results at one year.

## 4. Discussion

Masculine features of the upper third of the face include frontal bossing, a posteriorly displaced hairline, and heavier, straighter eyebrows. Facial feminization of this portion of the face typically involves bony contouring to reduce frontal bossing, brow lift, and lowering of the hairline. The implementation of forehead sculpturing in plastic surgery dates back to 1987 and has traditionally employed an open surgical technique via a coronal forehead flap or pretrichial approach [4]. While the open approach has been safe and efficacious, it may leave the patient with a cosmetically unappealing scar and may increase the risk for alopecia and forehead paresthesias [12].

A minimally invasive approach to the anterior table of the frontal sinus has become a useful option for the reduction of frontal sinus fractures in the trauma setting [8]. Endoscopic assistance allows magnified visualization with the benefit of smaller scars, reduced risk of nerve injury, decreased length of hospital stay, and less soft tissue dissection as compared to open techniques [10, 13]. Song et al. reported a sentinel case of endoscopic frontal bone contouring in a 42-year-old Korean woman in 1995 [14]. A recent retrospective report on the success of the endoscopic approach to correct female frontal bossing has lent support to the efficacy, decreased morbidity, and positive aesthetic outcomes. Additionally, endoscopic contouring can be performed under a local anesthetic leading to same day discharge and reduced costs [10].

Of significant importance in the successful outcome of endoscopic reduction of the anterior table is preoperative planning. Lateral cephalograms help assess the thickness of the anterior table and supraorbital ridge in relation to the frontal sinus in order to prevent penetration into the sinus. Males tend to have a more pronounced frontal sinus and are particularly at risk for sinus penetration [11]. Patients ideally suited for endoscopic correction are those with mild-moderate frontal bossing and adequate (>5 mm) anterior table thickness. Those with a thin anterior table are better suited for a traditional open frontal bone setback secured with miniplates or titanium mesh. Additionally, if the patient required a lowering of the hairline or a frontal brow lift, then an open approach is used.

Technical challenges of the operation do include a learning curve with regard to the use of the drill under limited access of the endoscopic ports. Entry into the frontal sinus must be avoided by continuously checking the degree of reduction and bone translucency by inspection and palpation [10]. The endoscopic approach is even more challenging when a nasofrontal angle correction is also desired. For example, orbital opening or burring of the frontomalar region cannot be accomplished endoscopically using incisions above the hairline. In these scenarios, an open approach can be used. Care must also be taken to protect the supraorbital and supratrochlear nerves during the dissection when using either technique [15].

Reduction rhinoplasty is also of chief importance in facial feminization surgery, especially in regard to the profile view. The stereotypical female nose has increased concavity at the dorsum and a wider nasofrontal angle with a slightly upturned nasal tip resulting in a more obtuse nasolabial angle. Thus, these areas are targeted to increase the nasolabial and nasofrontal angle. Additionally, the overall size of the nose should be in harmony with the rest of the face. This usually means contouring the nasal dorsum and tip to create a more delicate petite nose [16]. While the middle and lower thirds of the nose can be addressed without any concurrent operations, the radix and nasofrontal angle are more difficult to control without also altering the glabella and brow region. While a deep radix can be augmented and the nasofrontal angle softened via rhinoplasty alone, creation of a significantly widened nasofrontal angle can only be achieved with frontal bone reduction in combination with rhinoplasty [17]. We believe that, based on our operative experience, concurrent nasal and frontal bone feminization is not only safe but also a more powerful and convenient technique. We routinely see patients in consultation that are out of state, and, therefore, by reducing the number of operations for these patients, we are able to address their facial feminization needs more expeditiously. Furthermore, health care costs may also be better controlled. As these patients are now frequently covered for their procedures under insurance plans, limiting the number of hospital stays via concurrent operations is a benefit in that regard as well.

## 5. Conclusions

Gender affirmation surgery is a rapidly developing field that necessitates ongoing refinement. Patients typically require a multitude of reconstructive and medically necessary surgeries tailored to the patient's own needs. The feminization of facial features significantly impacts the mental health and quality of life of male-to-female transgender individuals. It has been shown that those who have undergone a transition without facial feminization surgery have significantly lower quality of life compared to those who have [16]. The success of such surgeries depends on a thorough understanding of the differences between the male and female skeletal contour and structure.

The open surgical approach to a reduction of frontal bossing has been described for many years in the male-to-female transgender literature with favorable outcomes. However, the use of the endoscopic approach for supraorbital rim reduction in facial feminization offers patients a less invasive option and is a novel indication in this patient population. Furthermore, combining reduction rhinoplasty with frontal brow reduction in a single operation is safe and efficacious with potentially reduced healthcare costs.
